# A brain imaging study of dopamine receptor D_2_ availability in cannabis dependent users after recovery from cannabis-induced psychosis

**DOI:** 10.3389/fpsyt.2023.1230760

**Published:** 2023-10-27

**Authors:** Aviv M. Weinstein

**Affiliations:** Department of Psychology and Behavioral Science, Ariel University, Ariel, Israel

**Keywords:** cannabis, psychosis, dopamine, D_2_, SPECT imaging

## Abstract

There is increased risk of psychosis associated with cannabis use disorder and the interaction of THC with dopamine neurotransmission is complex. It is important to investigate the recovery from cannabis-induced psychosis and its effects on the brain’s dopamine neurotransmission. This study was to evaluate dopamine receptor D_2_ availability in the striatum (caudate/putamen) in recently abstinent cannabis dependent users after recovery from psychosis in comparison with abstinent MDMA “ecstasy” abusers and healthy control participants. Participants were eight abstinent ex cannabis-dependent users who were treated for cannabis-induced psychosis with anti-psychotic medication and psychosocial support for 4 months in an inpatient treatment center for drug users. They were compared with nine abstinent ex MDMA “ecstasy” abusers who received medication and psycho-social treatment for 4 months at the same treatment facility and eight healthy control participants. All participants were scanned with bolus and constant infusion of [^123^I] Iodobenzamide (IBZM) in Single Photon Computed Tomography (SPECT). Cannabis abstinent users who were treated for cannabis-induced psychotic episodes showed no difference in dopamine D_2_ receptor availability in the caudate compared with abstinent MDMA “ecstasy” abusers and healthy control participants. This finding indicates minimal effects of cannabis-induced psychosis on dopamine reward mechanisms. There is evidence for reduced D_2_ receptor availability measures in the right putamen (uncorrected) which may indicate a residual effect of anti-psychotic medication.

## Introduction

There is growing evidence of high rates (40–50%) of substance use disorders among individuals with psychotic illness especially in young adolescents where it can be up to 70% (1, 2).

Cannabis use is a risk factor for developing schizophrenia although the issue is controversial (3, 4). A prospective study of cannabis use showed that cannabis use increased the risk for psychotic symptoms in young people (aged 14–24 years), particularly in individuals who are predisposed for psychosis (5). Pre-clinical studies showed that acute administration of Δ-9 tetrahydrocannabinol (THC) activated dopamine in the meso-limbic dopamine system and release of acetylcholine in the hippocampus and prefrontal cortex (6). Repeated daily administration of THC for 7 or 14 days reduced dopamine turnover in the medial prefrontal cortex (7). Consistent with this evidence, brain-imaging studies showed THC-induced dopamine release in the Striatum and limbic regions (8, 9). This evidence supports the notion of psychotogenic properties of THC and the hypothesis of dopamine over-activity in schizophrenia (10).

Laboratory experiments studied the relationship between cannabis and psychosis [see reviews by Radhakrishnan et al. (1), Ranganathan et al. (2), Sherif et al. (11), Volkow et al. (12), Hindley et al. (13)]. D’Souza et al. (14) showed that THC induced positive symptoms of schizophrenia. Morrison et al. (15) reported similar effects of 2.5 mg i.v of THC in healthy participants. Hindley et al. (13) have reviewed eligible studies on the acute administration of THC and four studies on CBD with THC administration. They have reported that THC increased total psychiatric symptom severity, positive symptom severity and negative symptom severity with a large effect sizes.

There is consistent evidence that THC acutely induces psychotic symptoms via CB_1_ receptor partial agonism and that heavy long-term cannabis use during adolescence exacerbates the risk of psychosis (16). Individuals with high risk for psychosis had high endocannabinoid levels in peripheral blood (17). Those with prodromal psychotic symptoms of a pre-psychotic phase or attenuated psychosis syndrome showed high activity of endocannabinoids during the beginning of the disorder (18). There is further evidence that chronic cannabis use leads to CB_1_ receptor down-regulation similarly to medication naive cannabis-free patients with schizophrenia (19). Furthermore, peripheral endocannabinoid anandamide (endogenous CB_1_ receptor agonist) is elevated in individuals with schizophrenia (20).

Cannabis exacerbates psychotic symptoms among individuals with schizophrenia (21) and there is evidence showing an association between psychosis and dopamine, thus elevated striatal dopamine synthesis and release capacity has been found in people with genetic and/or clinical high risk for schizophrenia in some studies (22). THC affects dopaminergic transmission with some consistent and complex findings (23), which merit further investigation.

Very few brain imaging studies investigated the link between cannabis use and psychosis. Delta-9-THC induced psychotic symptoms but no significant dopamine release in healthy volunteers, suggesting that dopamine release in the striatum is not responsible for cannabis-induced psychosis (24). This evidence is contrary to the argument that enhanced cannabis-induced dopamine release may give rise to delusions and hallucinations (10).

Regular cannabis users after abstinence and recovery display dopamine D_2_ measures of availability that is not different from healthy control participants (25–27). These findings indicate normal levels of dopamine function after recovery. However, recovery as result of treatment cannot be ascertained because baseline measures of dopamine D_2_ availability were not taken in these studies. Furthermore, it is not known whether recovery from cannabis-induced psychosis is associated with normal measures of dopamine D_2_ receptor availability. The increased risk of cannabis-induced psychosis and the complex interaction of THC with dopamine neurotransmission, merits an investigation on the effects of cannabis-induced psychosis on the brain’s dopamine neurotransmission mechanisms after recovery.

Previous studies have investigated the relationship between striatal dopamine function and symptoms in psychotic disorders, and they have measured the whole striatum (28–30). A recent study has measured spatial variability in dopamine synthesis capacity and psychotic symptoms combining ^18^F-DOPA in positron emission tomography (PET) and resting-state magnetic resonance imaging in patients with first-episode psychosis and healthy control participants (31). Although no subdivision relationships were found when using anatomical divisions, dopamine function in striatal areas connected to the default mode network correlated with negative symptoms. These findings suggest that individual differences in the topography of dopamine dysfunction within the striatum contribute to psychotic symptoms.

The aim of this study was to evaluate dopamine receptor D_2_ availability in the striatum (caudate/putamen) in abstinent cannabis users after recovery from cannabis-induced psychosis. We have also included a control group pf recently abstinent MDMA ‘ecstasy” abusers after 4–6 months of recovery and healthy control participants. The rationale for using this group is that MDMA or “ecstasy” abuse is associated with chronic effects on the brains serotonin 5-HT system but its effects on the brain’s dopamine neurotransmission during drug abuse and recovery is unknown. Due to difficulty in imaging cannabis and psycho-stimulant dependent individuals with history of psychosis under medication treatment, no baseline imaging measures were taken. These patients underwent psychiatric assessment and brain imaging after treatment. We hypothesized that abstinent cannabis users after recovery from cannabis-induced psychosis would show comparable dopamine D_2_ availability in the striatum to abstinent MDMA “ecstasy” abusers after 4–6 months of recovery and healthy control participants.

## Procedure

### Participants

Seventeen in-patient and 8 control participants were recruited for this study. This study was approved by the Institutional Review Board of Tel Aviv Sourasky Medical Center in Israel and informed consent was obtained from all participants. Participants were excluded for psychiatric disorders such as attention deficit hyperactivity disorder, taking medications that affect the CNS, neurological damage, infection that might the affect CNS (HIV, syphilis, and herpes), pregnancy or age under 18 years. All participants fulfilled the criterion of drinking less than 2 standard units of alcohol a day, drinking less than 3 cups of coffee a day and having a body mass index between 18.5 and 25, based on self-reports.

### Abstinent cannabis users who recovered from cannabis-induced psychosis

The group consisted of eight cannabis-dependent users, six males and two females with mean age 23 years and 4 months (S.D = 1.03) fulfilling DSM-IV (32) with diagnosis of substance-induced psychotic disorder (SIPD). They were treated with anti-psychotic medication and psychosocial support for 4 months in an inpatient treatment center. Their psychosis lasted on average for 1 month (S.D = 0.53). All participants used cannabis regularly before their psychosis. Five of them have also occasionally used psycho-stimulants such as MDMA, LSD and psilocybin but according to self-reports, they have not used psycho-stimulants during the month before their psychosis. All participants were in remission from substance induced psychosis. Psychotic symptoms were measured by a Psychiatrist. They were scanned between 2012 and 2016.

### Abstinent “ecstasy” abusers who recovered from “ecstasy” abuse

Nine abstinent ex MDMA “ecstasy” abusers, eight males and one female with mean age 25 years (S.D = 3.5). They fulfilled DSM-IV (32) diagnosis of substance abuse and dependence without substance-induced psychotic disorder (SUD). The abstinent MDMA “ecstasy” abusers took part in our earlier study (33) and underwent the same recruitment and assessment procedure as the main group of cannabis users who recovered from cannabis-induced psychosis and the same imaging procedure in the same scanner SPECT (Hawkeye, GE Healthcare). All participants used MDMA “ecstasy” regularly before treatment. According to their self-report they also used LSD, psilocybin, amphetamines and inhalants but they have not regularly used cocaine or heroin (see Table 1). They were not taking medication during scanning.

**Table 1:** Demographic and participants characteristics for each group.

	Abstinent cannabis users recovered from psychosis	Abstinent MDMA “ecstasy” abusers	Healthy Control participants	Comparison between abstinent cannabis users and control participants
Number and frequencies (male: female)	8 (6:2)	9 (8:1)	8 (7:1)	n.s.
Age- mean (S.D)	23.3 (1.03)	25 (3.5)	35.75 (6.5)	*t* (1,7) = 5.77; *p* = 0.001.
Years of education (S.D)	12 (1)	12 (0.9)	13.75 (1.6)	n.s
Drug use history				
Alcohol consumption units a week (S.D)	2 (3.2)	2.95 (2.54)	3 (2.6)	n.s
Nicotine cigarettes per day (S.D)	15.6 (7.8)	17 (6.75)	3.6 (6)	*F* (1,18) = 18.47; *p* < 0.001
Cannabis grams per day (S.D)	2 (3.7)	2 (4)	0	
Life time use of Hallucinogenic drugs (L.S.D)	<5	75 (80)	0	
MDMA “ecstasy”	<5	220	0	
Cocaine	0	25 (36)	0	
Amphetamines	0	2.8 (4)	0	
Inhalants	0	20.8 (62)	0	
Opiates- number of times used	0	2.4 (3)	0	
Psilocybin “Magic mushrooms”	0	9.4 (16)	0	

Frequencies (percentages), age: reported in years, Tobacco consumption; cigarettes per day, alcohol consumption habits; drink defined as glass of wine or 250 mL of beer or one shot of alcoholic beverages; education reported in years, drug use; cannabis or synthetic cannabis, significant level of difference between drug groups within the total sample; n.s.: non-significant difference.

### Healthy control participants

The control group consisted of eight healthy drug-free (based on self-report) participants, seven males and one female with mean age 35 years and 9 months (S.D = 6.5). They took part in our earlier study (33) and underwent the same recruitment and assessment procedure as the main group of cannabis users who recovered from cannabis-induced psychosis and the same imaging procedure in the same scanner SPECT (Hawkeye, GE Healthcare).

### Assessment procedure-questionnaires

#### Demographic and substance use history questionnaires

The demographic questionnaire included items on education level, age, and gender, use of psychoactive substances like cannabis and MDMA “ecstasy,” LSD and psilocybin, as well as tobacco, and alcohol.

#### Structured clinical interview for DSM-IV

##### Beck depression inventory

The Beck Depression Inventory (BDI) is a self-reported inventory measuring symptoms of depression (34). The inventory includes 21 items, each item is rated on a scale from 0 to 4 and a total score is computed by summing the items. The BDI demonstrates high internal consistency, with Cronbach internal reliability of α = 0.86 and 0.81 for psychiatric and non-psychiatric populations, respectively (35).

##### Spielberger State–Trait Anxiety Inventory

The Spielberger State–Trait Anxiety Inventory (STAI) is self-reported 40 items questionnaire; 20 items of trait anxiety inventory (A- Trait) and 20 items of state anxiety inventory (A-State) (36). Scores on a Likert scale range from 1 “not at all” to 4 “agree very much.” Total score is computed by summing the items, higher scores indicate greater trait or state anxiety. The STAI had been validated with average Cronbach internal reliability of *α* =0.88 (36).

##### Psychological treatment

Treatment of the main group included two sessions a week of individual psychotherapy, one family therapy session a week and daily sessions of group psychotherapy.

##### Pharmacological treatment

Cannabis users who were treated for cannabis-induced psychosis received pharmacological treatment by a Psychiatrist. They were treated with anti-psychotic medication- Risperidone 3–4 mg per day, Olanzapine 20 mg per day, 1 patient received Lithium 300–600 mg per day, and 2 patients received Clonazepam 0.5 mg per day. Medication was reduced gradually during treatment in accordance with patients’ recovery. A month after cessation of anti-psychotic medication the SPECT scan was performed. Time since last use of cannabis was between 4 and 6 months. Five of the abstinent MDMA “ecstasy” abusers were treated with antidepressant medication (Sertraline, Venlafaxine, Fluoxetine and Escitalopram) and six of them were treated with relaxants (Clonazepam and Diazepam). They were treated with medication and psychosocial support for 4 months in an inpatient treatment centers for drug users. All patients were scanned a month after treatment when they were not taking medication and they were abstinent from drugs based on urine samples. The month after treatment time-point was in order to ensure that there are no residual medication effects that may affect scanning. The patients were not symptomatic at the time of scanning and that was verified by a Psychiatrist.

### Imaging procedure

All participants have filled in a consent form a week before the study. They have fasted for 2 h in the morning before scanning. In the morning of the study they have received Iodine (Lugol). Participants have been admitted to the hospital ward at 10 am. They were not allowed to eat or drink anything but water and they were allowed to go to the bathroom when needed. Starting at 10:30 a.m., they received a bolus injection of 5–6 mCi of [^123^I] IBZM in Single Photon Computed Tomography (SPECT) (Hawkeye, GE Healthcare), followed by constant infusion of 5–6 mCi of [^123^I] IBZM (1.7–2 mCi/h). for 3 hours while resting on a hospital bed and another 50 min during baseline scan following procedure described by Laruelle et al. (37).

[^123^I] IBZM with specific activity >5,000 Ci/mmol and radiochemical purity >95% supplied by Eldan Medical equipment. [^123^I] IBZM is a dopamine D_2_ antagonist radiotracer for imaging dopamine *in vivo* in SPECT. The protocol of administration (bolus plus constant infusion) induces a state of sustained binding equilibrium in the absence of pharmacological or behavioral challenge (38). After a baseline SPECT scan in which constant infusion was maintained they returned to their room and were released from the study.

### Image analysis

All groups of participants underwent the same image analysis procedure reported by Weinstein (33). A measure of dopamine receptor availability binding potential (BP_ND_) can be calculated by the equation BP_ND_ = (S−O)/O where S and O are activity concentrations in the striatum and occipital cortex, respectively, under equilibrium conditions (37). All images were registered and normalized to an IBZM template (39) using the pre-processing tools of Statistical Parametric Mapping (SPM),^1^ implemented on a Matlab platform. Volume of interest (VOI) analysis image comparisons were performed using the MarsBaR tool within SPM^2^ described in Tzourio-Mazoyer et al. (40). VOIs, including the putamen, caudate nucleus, and the occipital lobe of each image were defined on the decay-corrected [^123^IBZM] images. For each scan acquisition, alignment of the image frames was checked. Since only minimal head movements were observed over the acquisitions, no correction for movement was performed. The binding potential (BPnd) described above was then calculated for right and left side caudate and putamen for each patient scan.

A second volume of interest (VOI) analysis was performed using the Xeleris software of GE. SPECT data were analyzed blind to the diagnosis. Count projections were pre-filtered using the Wiener 0.5 filter. The four slices with the highest striatal uptake were summed and were attenuation-corrected using the Chang method of attenuation correction. Standard region of interest templates of the striatum and occipital cortex were used as described by Lokkegaard et al. (41). Striatal specific binding was calculated as the ratio described earlier. Since the results using SPM were more accurate and reproducible the second analysis of VOI will not be presented here.

[^123^I] IBZM SPECT imaging using the bolus injection and a single scan at 90 min post injection is a reproducible method showing acceptable test–retest variability and reliability (42). A comparison of striatal D2 receptor occupancy measured by [^123^I] IBZM SPECT or [^11^C] raclopride binding potential in treated schizophrenic patients showed that although anatomical resolution was superior in PET, D_2_ availability almost perfectly correlated between both methods (43).

### Statistical analysis

Measures of BPnd for right and left side caudate and putamen for each scan were calculated using paired one-way ANOVA tests.

## Results

### Drug and alcohol use and questionnaire ratings

One female patient who recovered from psychosis was excluded from analysis due to abnormally low binding potential BPnd measures. Table 1 describes demographic data and drug use history in all participants.

Abstinent cannabis users scored on STAI (A- Trait) = 38.13 (SD = 10.92), STAI (A- State) = 37.88 (S.D = 12.25) BDI = 7.88 (S.D = 7.86). Control participants scored on STAI (A- Trait) = 37.88 (S.D = 12.25) STAI (A- State) = 34.25 (S.D = 8.06) BDI = 3.25 (S.D = 4.72). There was no significant difference between the abstinent cannabis users and the healthy control participants group in STAI *t* (1, 14) = 1.61; *p* = n.s SSAI t (1, 14) = 0.855; *p* = n.s or BDI scores *t* (1, 14) = 1.6; p = N.S. There was no significant difference between the abstinent cannabis users and abstinent “ecstasy” abusers in STAI *t* (1, 14) = 0.68; *p* = 0.52 SSAI *t* (1, 14) = 0.976; *p* = 0.36 or BDI scores *t* (1, 14) = 1.17; *p* = 0.28. The groups showed no significant difference in alcohol consumption measures but both abstinent groups smoked nicotine cigarettes per day more than control participants.

### Measures of receptor availability (BPnd)

Table 2 shows binding potential BPnd measures in all participants.

**Table 2:** Dopamine D_2_ receptor binding potential (BP_ND_) measures in abstinent cannabis users, abstinent MDMA “ecstasy” abusers and healthy control participants.

Subject Number	Abstinent cannabis users				Abstinent “ecstasy” abusers				Control participants			
	Left putamen	Right putamen	Left caudate	Right caudate	Left putamen	Right Putamen	Left caudate	Right Caudate	Left putamen	Right putamen	Left caudate	Right caudate
1	0.37	0.37	0.53	0.45	0.84	0.99	0.5	0.5	1.71	2.03	1.29	1.55
2	0.68	0.68	0.72	0.72	0.72	0.71	0.43	0.62	0.5	0.82	0.69	0.82
3	0.82	0.82	0.92	0.88	0.62	0.76	0.29	0.59	0.66	0.83	0.56	0.47
4	1.07	1.07	1.02	1.01	0.53	0.57	0.28	0.42	0.89	0.84	0.61	0.65
5	0.38	0.38	0.48	0.42	0.68	0.78	0.31	0.45	0.69	0.78	0.46	0.5
6	1.06	1.06	0.81	0.83	1.11	1.06	0.73	0.77	1.01	1.07	0.33	0.5
7	0.34	0.34	0.27	0.27	1.34	1.39	0.98	0.92	1.36	1.49	0.71	0.89
8	0.74	0.74	0.83	0.78	0.69	1.1	0.69	0.66	0.8	0.91	0.46	0.62
9					0.69	0.83	0.53	0.52				
Mean	0.68	0.68	0.70	0.67	0.80	0.91	0.53	0.61	0.95	1.10	0.64	0.75
STDEV	0.30	0.30	0.25	0.26	0.26	0.25	0.24	0.16	0.40	0.44	0.29	0.36

Table 3 shows comparisons between in D_2_ binding potential BP_ND_ measures in the caudate and putamen in all participants. There were no differences in dopamine D_2_ receptor availability in the caudate between abstinent cannabis users compared with abstinent MDMA “ecstasy” abusers and healthy control participants. Using a simple comparison with one-way ANOVA, abstinent cannabis users had lower right putamen BP_ND_ measures compared with control participants *F* (1, 14) = 4.80, *p = 0.046. When comparing abstinent cannabis users with abstinent MDMA “ecstasy” abusers and healthy control participants using one-way ANOVA with Bonferroni corrections the difference has become non-significant *F* (2, 23) = 2.91 *p* = 0.076. When comparing the cannabis group with abstinent MDMA “ecstasy” abusers and healthy control participants using one-way ANOVA with Bonferroni corrections none of the other areas have shown significant group differences: Left putamen *F* (2, 23) = 1.345, *p* = 0.28, Left caudate *F* (2, 23) = 0.86, *p* = 0.44, Right caudate *F* (2, 23) = 0.497, *p* = 0.62.

**Table 3:** A comparison of D_2_ binding potential BP_ND_ measures in the Caudate and putamen (left and right) between abstinent cannabis users after recovery from psychosis, abstinent drug users and healthy control participants (one-way ANOVA).

	Left putamen	Right putamen	Left caudate	Right caudate
abstinent cannabis users vs. control participants	*F* (1,14) = 2.33, *p* = 0.15	*F* (1,14) = 4.80, *p = 0.046	*F* (1,14) = 0.19, *p* = 0.67	*F* (1,14) = 0.26, *p* = 0.62
abstinent MDMA “ecstasy” abusers vs. control participants	*F* (1,15) = 0.86, *p* = 0.37	*F* (1,15) = 1.18 *p* = 0.30	*F* (1,15) = 0.76 *p* = 0.40	*F* (1,15) = 0.12 p = 0.30
abstinent cannabis users vs. abstinent MDMA “ecstasy” abusers	*F* (1,15) = 0.51 *p* = 0.49	*F* (1,15) = 1.79, *p* = 0.21	*F* (1,15) = 1.46 *p* = 0.25	*F* (1,15) = 0.01 *p* = 0.76

*p < 0.05.

Dopamine D_2_ availability was within normal range of 0.3–2.5 (44).

**Figure 1:**
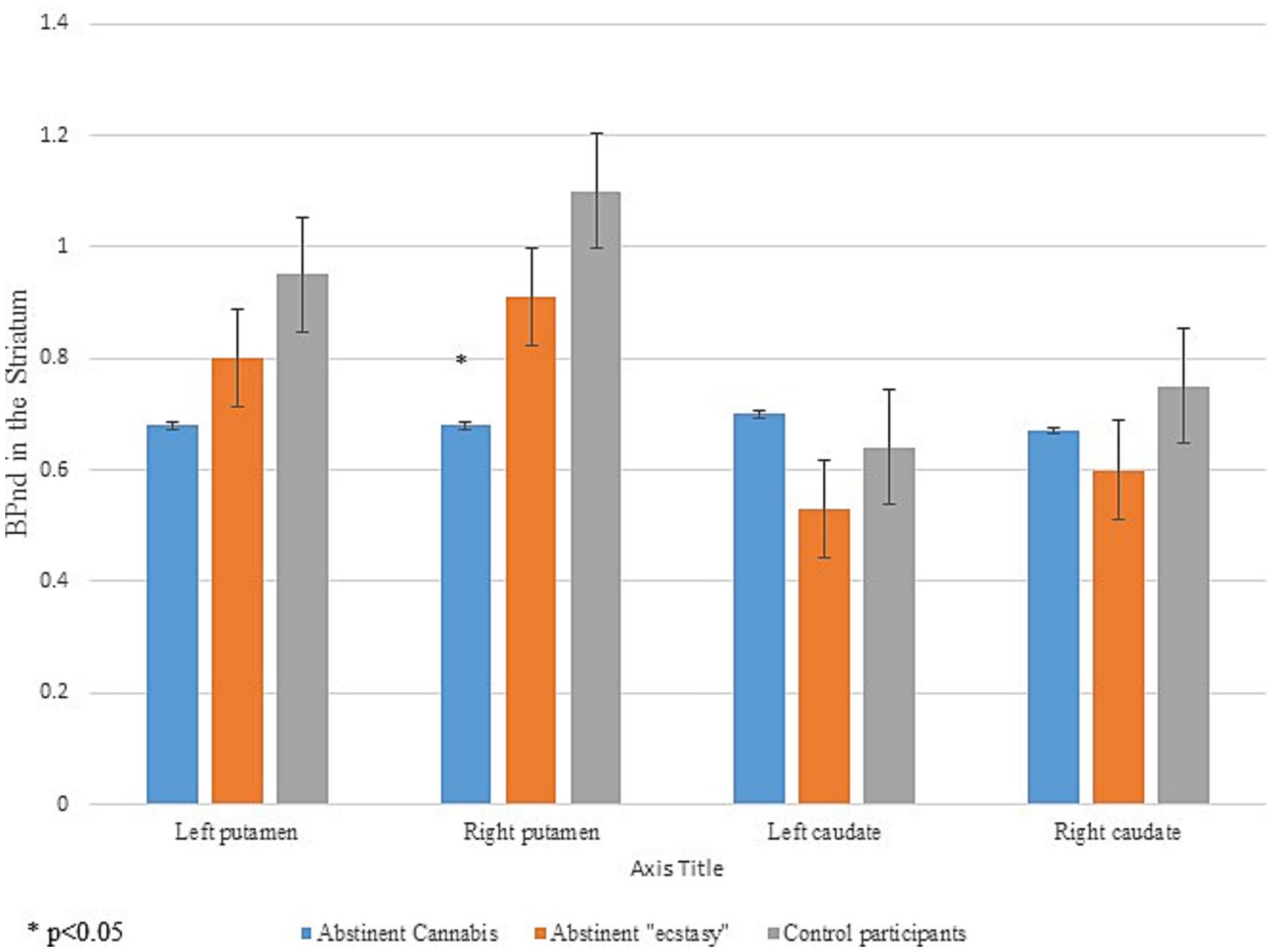
Dopamine receptor D_2_ binding potentials in the striatum in all participants.

Figure 1 shows D_2_ binding potential BP_ND_ measures in the striatum (left and right caudate and putamen) in all participants.

## Discussion

There is a controversy whether using cannabis regularly is posing a risk for psychotic disorders. Adolescent cannabis use was associated with psychosis in a longitudinal study (5). This association could be explained by causality, interactions between genes and environment, shared etiology, or self-medication (45, 46). The age of the beginning of cannabis use correlated with the age at onset of psychosis (45, 47, 48). Also, individuals who used cannabis frequently during adolescence were at greater risk for psychosis and schizophrenia (47, 49–53). Cannabis use is estimated to increase the risk of schizophrenia particularly among those using high THC potency (49, 54).

There are several possible biological mechanisms that may underlie cannabis induced-psychosis. The dopaminergic system has been for a long time considered to play an important role in psychotic disorders, but there is increasing evidence that the cannabinoid system may also be involved. High levels of anandamide, an endogenous cannabinoid agonist, were detected in the cerebrospinal fluid of persons with schizophrenia (55). Additionally, persons with schizophrenia had a greater density of CB_1_ receptors in the prefrontal cortex than control participants (55). Cannabis use interacts with the dopamine catechol-O-methyl transferase (COMT) Val158Met polymorphism (56). Finally, regular cannabis use exacerbated the symptoms among recent onset cases of schizophrenia (57, 58). However, most cannabis users do not develop schizophrenia. Cannabis may initiate psychotic symptoms in individuals with genetic vulnerability and family history of mental illness, and this may cause concern among healthcare professionals.

We report the first study to the best of our knowledge that has assessed dopamine D_2_ receptor availability in abstinent cannabis dependent individuals who recovered from cannabis-induced psychosis. Their measures of D_2_ receptor availability in the caudate were not different between abstinent MDMA “ecstasy” abusers and healthy control participants. This evidence is compatible with previous studies measuring D_2_ receptor availability in recovered cannabis-dependent users. The length of abstinence of participants was 15 weeks (25), 4 weeks (27) and 18 months (26). Most of the imaging evidence (dopamine imaging and other methods) points to normalization of function following abstinence and so these findings are entirely in keeping with that literature. However, we have used a different methodology from previous studies by using a different radio ligand and scanner (IBZM in SPECT vs. [11C] raclopride in PET). Furthermore, our patients were tested after recovery from cannabis-induced psychosis whereas previous studies included current cannabis users.

The lack of differences in D_2_ receptor availability may be due to abstinence and the adaptive changes that occur after a prolonged period of abstinence. Stokes et al. (59) argued that cannabis use history is not related to changes in striatal dopamine D_2_ receptor availability. Urban et al. (27) maintained that the effects of THC are mediated by the endocannabinoid system and that striatal DA neurotransmission is not changed in cannabis dependence. This is supported by evidence for a reversal or normalization of CB_1_ receptor within a few weeks of abstinence in chronic cannabis users, using the novel CB_1_ receptor-selective radio ligand [^18^F] FMPEP-d2 in PET (60). Alternatively, differences in striatal DA transmission in cannabis users compared with healthy control participants may have resolved during the abstinence phase as shown in our study.

Our findings are compatible with those reported by Bloomfield et al. (61) of no association between cannabis-induced psychotic symptoms and dopamine synthesis capacity. Furthermore, striatal dopamine release was reduced after amphetamine challenge in cannabis users (62, 63), although these studies reported reduced dopamine release in active cannabis users. Likewise, Leroy et al. (64) reported reduced DAT availability in cannabis users and Albrecht et al. (65) showed that D_2_ receptor availability was associated with current cannabis use. These findings as well suggest that reduced dopamine activity depends on active cannabis use.

Barkus et al. (24) showed that positive and general symptoms on the Positive and Negative Syndrome Scale (PANSS) increased at 30 min following THC administration THC has not induced dopamine release in the striatum measured with ^123^I-iodobenzamide ([^123^I] IBZM) in SPECT. Secondly, positive psychotic symptoms and DA release were unrelated. They argued that their findings do not support a central role for striatal DA in THC-elicited psychosis. Their results are contrary to the results of two studies that showed significant dopamine release following THC ingestion in healthy volunteers. THC reduced [^11^C] raclopride binding the ventral striatum and the pre-commissural dorsal putamen but not in other striatal sub-regions in healthy participants in PET (9). THC administration also induced a significant reduction in [^11^C] raclopride binding in the limbic striatum in a large group of healthy volunteers (8). Although THC induces an increase in dopamine release in the striatum, it is not known precisely how cannabis induces psychotic symptoms. It is plausible that these symptoms are a result of cannabis-induced dopamine dysregulation (10) or its effects on CB_1_ receptors, Glutamate or GABA.

Previous studies showed the effects of anti-psychotic treatment on the putamen. Farde et al. (66) reported that clinically effective doses of chemically distinct neuroleptic drugs result in 85 to 90 percent occupancy of D_2_ dopamine receptors in the putamen of schizophrenic patients using [^11^C] raclopride in PET. These findings indicate that the effects of anti-psychotic medication during treatment of cannabis-induced psychosis may have been evident in the putamen. It is plausible that these effects extended beyond the 3 months of treatment and hence the reduced availability of D_2_ in the right putamen after recovery in our study.

This study also showed comparable D_2_ availability in the caudate and putamen measures in abstinent MDMA “ecstasy” abusers and healthy control participants. MDMA (“ecstasy”) operates through its binding affinity to the serotonin receptors (67). MDMA also binds to the serotonin transporter (SERT), thus prolonging signaling at the synapses. Little is known about the effects of chronic MDMA ‘ecstasy” use on the dopamine reward mechanisms in humans.

Recent studies have shown that use of highly potent and rewarding novel psychoactive substances (NPS) is associated with high rates of psychosis and 25% of first-episode psychoses are substance-induced psychosis (68). Ricci et al. (69) have reported that first-episode psychotic patients (FEPp) using cannabis showed higher levels of positive symptoms, dissociative experiences and worse function than their non-user counterpart, which persist after 8 months. Ricci et al. (70) have reported that THC-users, especially synthetic cannabinoid users (SCs) showed more severe positive symptoms than non-users and worse recovery after 9 months. Martinotti et al. (68) have proposed a new diagnosis of substance-related exogenous psychosis (SREP) which refers to both transient and persistent psychoses associated with substance use which is distinct from schizophrenia. Finally, there is evidence that rTMS can be effective in the treatment of addiction, with promising results in treatment of cocaine, and cannabis use disorder (71). Future studies could examine the use of rTMS in treatment of patients with cannabis use disorder and those with cannabis-induced psychosis.

### Limitations

First, this study is a cross-sectional study hence it is not possible to ascertain directly recovery from cannabis induced psychosis and the effects of medication on the brain’s dopamine D_2_ receptor availability. Secondly, no baseline measures of D_2_ receptor availability were taken since cannabis users were admitted in an acute psychotic state when it was not possible for scanning. Although all patients were assessed by a Psychiatrist, during treatment and recovery, no measures of psychotic symptoms are available. Third, both abstinent groups were younger than the control group and smoked more nicotine cigarettes per day and that may have affected the results. Furthermore, there is absence of a qualitative assessment of the study and image realignment correction was not performed. Our analysis methods were not able to use sub-divisions of the striatum for image analysis (apart from caudate-putamen). Finally, this was a relatively small sample of participants due to major difficulties recruiting and scanning patients who were treated for cannabis-induced psychosis and “ecstasy” abusers. According to our power calculations 15 participants in each group would be required in order to provide definite results. Unfortunately, most studies that measured dopamine occupancy in cannabis use disorder have used a smaller number of participants which is a limitation in these kind of studies due to recruitment issues.

## Conclusion

This study showed no difference in dopamine D_2_ receptor availability in the caudate between the abstinent cannabis users after recovery from cannabis-induced psychosis, abstinent MDMA “ecstasy” abusers and healthy control participants indicating minimal effects of cannabis-induced psychosis and chronic MDMA “ecstasy” abuse on dopamine reward mechanisms. Due to the small number of patients there is a possibility of type 2 error and the results should be regarded as preliminary and require further replication in larger samples. The findings suggest that remission of cannabis-induced psychosis is not associated with hyper-dopaminergic activity. This could either be because it has resolved. The lower D_2_ receptor availability measures in the right putamen (uncorrected) may indicate residual effect of anti-psychotic medication.

## Data availability statement

The raw data supporting the conclusions of this article will be made available by the authors, without undue reservation.

## Ethics statement

The studies involving humans were approved by Tel Aviv Sourasky Medical Center, Tel Aviv Israel. The studies were conducted in accordance with the local legislation and institutional requirements. The participants provided their written informed consent to participate in this study.

## Author contributions

The author confirms being the sole contributor of this work and has approved it for publication.

## Footnotes

^1^http://www.fil.ion.ucl.ac.uk/spm/software/spm2/

^2^http://marsbar.sourceforge.net

## References

[1] Radhakrishnan R Wilkinson ST D'Souza DC. Gone to pot - a review of the association between Cannabis and psychosis. Front Psych, 22. (2014) 5:54. doi: 10.3389/fpsyt.2014.00054. eCollection 2014, PMID: 24904437 PMC4033190

[2] Ranganathan M Skosnik PD Gupta S Cahill J Cortes-Briones J Sherif M. Cannabinoids and psychosis. Curr Pharm Des. (2016) 22:6380–91. doi: 10.2174/1381612822666160826105628 27568729

[3] Hall W. Is cannabis use psychotogenic? Lancet. (2006) 367:193–5. doi: 10.1016/S0140-6736(06)68012-4, PMID: 16427475

[4] Henquet C Van Os J. The coherence of the evidence linking cannabis with psychosis. Psychol Med. (2008) 38:461-2; author reply 462-4. doi: 10.1017/S0033291707002279, PMID: 18298878

[5] Henquet C Krabbendam L Spauwen J Kaplan C Lieb R Wittchen HU. Prospective cohort study of cannabis use, predisposition for psychosis, and psychotic symptoms in young people. BMJ. (2005) 330:11. doi: 10.1136/bmj.38267.664086.63, PMID: 15574485 PMC539839

[6] Pisanu A Acquas E Fenu S Di Chiara G. Modulation of Delta(9)-THC induced increase of cortical and hippocampal acetylcholine release by microopioid and D(1) dopamine receptors. Neuropharmacology. (2006) 50:661–70. doi: 10.1016/j.neuropharm.2005.11.023, PMID: 16427098

[7] Verrico CD Jentsch JD Roth RH. Persistent and anatomically selective reduction in prefrontal cortical dopamine metabolism after repeated intermittent cannabinoid administration to rats. Synapse. (2003) 49:61–6. doi: 10.1002/syn.10215, PMID: 12710016

[8] Bossong MG Mehta MA Berckel BN Howes OD Kahn RS Stokes PR. Further human evidence for striatal dopamine release induced by administration of ∆9-tetrahydrocannabinol (THC): selectivity to limbic striatum. Psychopharmacol (Berl). (2015) 232:2723–9. doi: 10.1007/s00213-015-3915-0, PMID: 25801289 PMC4816196

[9] Bossong MG van Berckel BN Boellaard R Zuurman L Schuit RC Windhorst AD. Delta 9-tetrahydrocannabinol induces dopamine release in the human striatum. Neuropsychopharmacology. (2009) 34:759–66. doi: 10.1038/npp.2008.138 18754005

[10] Kuepper R Morrison PD van Os J Murray RM Kenis G Henquet C. Does dopamine mediate the psychosis-inducing effects of cannabis? A review and integration of findings across disciplines. Schizophrenia Res. (2010) 121:107–17. doi: 10.1016/j.schres.2010.05.031, PMID: 20580531

[11] Sherif M Radhakrishnan R D'Souza DC Ranganathan M. Human laboratory studies on cannabinoids and psychosis. Biol Psychiatry. (2016) 79:526–38. doi: 10.1016/j.biopsych.2016.01.011 26970363

[12] Volkow ND Swanson JM Evins AE DeLisi LE Meier MH Gonzalez R. Effects of Cannabis use on human behavior, including cognition, motivation, and psychosis: a review. JAMA Psychiatry. (2016) 73:292–7. doi: 10.1001/jamapsychiatry.2015.3278, PMID: 26842658

[13] Hindley G Beck K Borgan F Ginestet CE McCutcheon R Kleinloog D. Psychiatric symptoms caused by cannabis constituents: a systematic review and meta-analysis. Lancet Psychiatry. (2020) 7:344–53. doi: 10.1016/S2215-0366(20)30074-2, PMID: 32197092 PMC7738353

[14] D'Souza DC Perry E MacDougall L Ammerman Y Cooper T Wu YT. The psychotomimetic effects of intravenous delta-9-tetrahydrocannabinol in healthy individuals: implications for psychosis. Neuropsychopharmacology. (2004) 29:1558–72. doi: 10.1038/sj.npp.1300496, PMID: 15173844

[15] Morrison PD Zois V McKeown DA Lee TD Holt DW Powell JF. The acute effects of synthetic intravenous Delta9-tetrahydrocannabinol on psychosis, mood and cognitive functioning. Psychol Med. (2009) 39:1607–16. doi: 10.1017/S0033291709005522, PMID: 19335936

[16] Colizzi M Ruggeri M Bhattacharyya S. Unraveling the intoxicating and therapeutic effects of Cannabis ingredients on psychosis and cognition. Front Psychol. (2020) 11:833. doi: 10.3389/fpsyg.2020.00833, PMID: 32528345 PMC7247841

[17] Appiah-Kusi E Wilson R Colizzi M Foglia E Klamerus E Caldwell A. Childhood trauma and being at-risk for psychosis are associated with higher peripheral endocannabinoids. Psychol Med. (2020) 50:1862–71. doi: 10.1017/S0033291719001946, PMID: 31422779

[18] Fusar-Poli P Borgwardt S Bechdolf A Addington J Riecher-Rössler A Schultze-Lutter F. The psychosis high-risk state: a comprehensive state-of-the-art review. JAMA Psychiatry. (2013) 70:107–20. doi: 10.1001/jamapsychiatry.2013.269, PMID: 23165428 PMC4356506

[19] Borgan F Laurikainen H Veronese M Marques TR Haaparanta-Solin M Solin O. In vivo availability of cannabinoid 1 receptor levels in patients with first-episode psychosis. JAMA Psychiatry. (2019) 76:1074–84. doi: 10.1001/jamapsychiatry.2019.1427, PMID: 31268519 PMC6613300

[20] Borgan F Kokkinou M Howes O. The cannabinoid CB1 receptor in schizophrenia. Biol Psychiatry: Cogn Neurosci Neuroimaging. (2021) 6:646–59. doi: 10.1016/j.bpsc.2020.06.018 33077399

[21] D'Souza DC Sewell RA Ranganathan M. Cannabis and psychosis/schizophrenia: human studies. Eur Arch Psychiatry Clin Neurosci. (2009) 259:413–31. doi: 10.1007/s00406-009-0024-2, PMID: 19609589 PMC2864503

[22] Howes OD Shatalina E. Integrating the neurodevelopmental and dopamine hypotheses of schizophrenia and the role of cortical excitation-inhibition balance. Biol Psychiatry. (2022) 92:513. doi: 10.1016/j.biopsych.2022.06.017, PMID: 36008036

[23] Bloomfield MA Ashok AH Volkow ND Howes OD. The effects of Δ^9^-tetrahydrocannabinol on the dopamine system. Nature. (2016) 539:369–77. doi: 10.1038/nature20153, PMID: 27853201 PMC5123717

[24] Barkus E Morrison PD Vuletic D Dickson JC Pilowsky LS Brenneisen R. Does intravenous Δ9-tetrahydrocannabinol increase dopamine release? A SPET study. J Psychopharmacol. (2011) 25:1462–8. doi: 10.1177/0269881110382465, PMID: 20851843

[25] Sevy S Smith GS Ma Y Dhawan V Chaly T Kingsley PB. Cerebral glucose metabolism and D2/D3 receptor availability in young adults with cannabis dependence measured with positron emission tomography. Psychopharmacol (Berlin). (2008) 197:549–56. doi: 10.1007/s00213-008-1075-1, PMID: 18270689 PMC5646272

[26] Stokes PR Mehta MA Curran HV Breen G Grasby PM. Can recreational doses of THC produce significant dopamine release in the human striatum? NeuroImage. (2009) 48:186–90. doi: 10.1016/j.neuroimage.2009.06.029 19539765

[27] Urban NB Slifstein M Thompson JL Xu X Girgis RR Raheja S. Dopamine release in chronic cannabis users: a [(11)C] raclopride positron emission tomography study. Biol Psychiatry. (2012) 71:677–83. doi: 10.1016/j.biopsych.2011.12.018, PMID: 22290115 PMC3314125

[28] Abi-Dargham A Gil R Krystal J Baldwin RM Seibyl JP Bowers M. Increased striatal dopamine transmission in schizophrenia: confirmation in a second cohort. Am J Psychiatry. (1998) 155:761–7. doi: 10.1176/ajp.155.6.761, PMID: 9619147

[29] Demjaha A Murray RM McGuire PK Kapur S Howes OD. Dopamine synthesis capacity in patients with treatment-resistant schizophrenia. Am J Psychiatry. (2012) 169:1203–10. doi: 10.1176/appi.ajp.2012.12010144, PMID: 23034655

[30] Jauhar S Veronese M Nour MM Rogdaki M Hathway P Turkheimer FE. Determinants of treatment response in first-episode psychosis: an ^18^F-DOPA PET study. Mol Psychiatry. (2019) 24:1502–12. doi: 10.1038/s41380-018-0042-4, PMID: 29679071 PMC6331038

[31] McCutcheon RA Jauhar S Pepper F Nour MM Rogdaki M Veronese M. The topography of striatal dopamine and symptoms in psychosis: an integrative positron emission tomography and magnetic resonance imaging study. Biol Psychiatry Cogn Neurosci Neuroimaging. (2020) 5:1040–51. doi: 10.1016/j.bpsc.2020.04.004, PMID: 32653578 PMC7645803

[32] American Psychiatric Association. Diagnostic and statistical manual of mental disorders. 4th ed. Washington, DC: American Psychiatric Association (1994).

[33] Weinstein A. Computer and video game addiction. Am J Drug Alcohol Abuse. (2010) 36:268–76. doi: 10.3109/00952990.2010.491879, PMID: 20545602

[34] Beck AT. Depression inventory. Philadelphia: Center for Cognitive Therapy (1978).

[35] Beck AT Steer RA Garbin MG. Psychometric properties of the Beck depression inventory: twenty-five years of evaluation. Clin Psychol Rev. (1988) 8:77–100. doi: 10.1016/0272-7358(88)90050-5

[36] Spielberger CD Gorsuch RL Lushene R Vagg PR Jacobs GA. Manual for the state-trait anxiety inventory. Palo Alto, CA: Consulting Psychologists Press (1983).

[37] Laruelle M Abi-Dargham A Van Dyck CH Rosenblatt W Zea-Ponce Y Zoghbi SS. SPECT imaging of striatal dopamine release after amphetamine challenge. J Nucl Med. (1995) 36:1182–90. PMID: 7790942

[38] Laruelle M Abi-Dargham A Van Dyck CH Gil R D’Souza CD Erdos J. Single photon emission computerised tomography imaging of amphetamine-induced dopamine release in drug-free schizophrenic subjects. Proc Natl Acad Sci U S A. (1996) 93:9235–40. doi: 10.1073/pnas.93.17.9235, PMID: 8799184 PMC38625

[39] Buchert R Berding G Wilke F Martin B von Borczyskowski D Mester J. IBZM tool: a fully automated expert system for the evaluation of IBZM SPECT studies. Eur J Nuc Med Mol Imaging. (2006) 33:1073–83. doi: 10.1007/s00259-006-0067-9, PMID: 16614812

[40] Tzourio-Mazoyer N Landeau B Papathanassiou D Crivello F Etard O Delcroix N. Automated anatomical labeling of activations in SPM using a macroscopic anatomical parcellation of the MNI MRI single-subject brain. NeuroImage. (2002) 15:273–89. doi: 10.1006/nimg.2001.0978, PMID: 11771995

[41] Lokkegaard A Werdelin LM Friberg L. Clinical impact of diagnostic SPET investigations with a dopamine re-uptake ligand. Eur J Nucl Med. (2002) 29:1623–9. doi: 10.1007/s00259-002-0938-7, PMID: 12458397

[42] Catafau AM Bullich S Danús M Penengo MM Cot A Abanades S. Test-retest variability and reliability of 123I-IBZM SPECT measurement of striatal dopamine D2 receptor availability in healthy volunteers and influence of iterative reconstruction algorithms. Synapse. (2008) 62:62–9. doi: 10.1002/syn.20465, PMID: 17960766

[43] Catafau AM Suarez M Bullich S Llop J Nucci G Gunn RN. Within-subject comparison of striatal D2 receptor occupancy measurements using [123I]IBZM SPECT and [11C]Raclopride PET. NeuroImage. (2009) 46:447–58. doi: 10.1016/j.neuroimage.2009.02.005, PMID: 19233294

[44] Wulff S Pinborg LH Svarer C Jensen LT Nielsen MØ Allerup P. Striatal D(2/3) binding potential values in drug-Naïve first-episode schizophrenia patients correlate with treatment outcome. Schizophr Bull. (2015) 41:1143–52. doi: 10.1093/schbul/sbu220, PMID: 25698711 PMC4535636

[45] Large M Sharma S Compton MT Slade T Nielssen O. Cannabis use and earlier onset of psychosis: a systematic meta-analysis. Arch Gen Psychiatry. (2011) 68:555–61. doi: 10.1001/archgenpsychiatry.2011.5 21300939

[46] Miettunen J Törmänen S Murray GK Jones PB Mäki P Ebeling H. Association of cannabis use with prodromal symptoms of psychosis in adolescence. Br J Psychiatry. (2008) 192:470–1. doi: 10.1192/bjp.bp.107.045740 18515902

[47] Di Forti M Sallis H Allegri F Trotta A Ferraro L Stilo SA. Daily use, especially of high-potency cannabis, drives the earlier onset of psychosis in cannabis users. Schizophr Bull. (2014) 40:1509–17. doi: 10.1093/schbul/sbt181, PMID: 24345517 PMC4193693

[48] Galvez-Buccollini JA Proal AC Tomaselli V Trachtenberg M Coconcea C Chun J. Association between age at onset of psychosis and age at onset of cannabis use in non-affective psychosis. Schizophr Res. (2012) 139:157–60. doi: 10.1016/j.schres.2012.06.007, PMID: 22727454 PMC3415971

[49] Andréasson S Allebeck P Engström A Rydberg U. Cannabis and schizophrenia: a longitudinal study of Swedish conscripts. Lancet. (1987) 2:1483–6. PMID: 2892048 10.1016/s0140-6736(87)92620-1

[50] Arseneault L Cannon M Poulton R Murray R Caspi A Moffitt TE. Cannabis use in adolescence and risk for adult psychosis: longitudinal prospective study. BMJ. (2002) 325:1212–3. doi: 10.1136/bmj.325.7374.1212, PMID: 12446537 PMC135493

[51] Di Forti M Marconi A Carra E Fraietta S Trotta A Bonomo M. Proportion of patients in South London with first-episode psychosis attributable to use of high potency cannabis: a case-control study. Lancet Psychiatry. (2015) 2:233–8. doi: 10.1016/S2215-0366(14)00117-5, PMID: 26359901

[52] Di Forti M Morgan C Dazzan P Pariante C Mondelli V Marques TR. High-potency cannabis and the risk of psychosis. Br J Psychiatry. (2009) 195:488–91. doi: 10.1192/bjp.bp.109.064220, PMID: 19949195 PMC2801827

[53] Zammit S Allebeck P Andreasson S Lundberg I Lewis G. Self reported cannabis use as a risk factor for schizophrenia in Swedish conscripts of 1969: historical cohort study. BMJ. (2002) 325:1199. doi: 10.1136/bmj.325.7374.1199, PMID: 12446534 PMC135490

[54] van Os J Bak M Hanssen M Bijl RV de Graaf R Verdoux H. Cannabis use and psychosis: a longitudinal population-based study. Am J Epidemiol. (2002) 156:319–27. doi: 10.1093/aje/kwf043, PMID: 12181101

[55] Dean B Sundram S Bradbury R Scarr E Copolov D. Studies on [3H]CP-55940 binding in the human central nervous system: regional specific changes in density of cannabinoid-1 receptors associated with schizophrenia and cannabis use. Neuroscience. (2001) 103:9–15. doi: 10.1016/s0306-4522(00)00552-2, PMID: 11311783

[56] Caspi A Moffitt TE Cannon M. Moderation of the effect of adolescent-onset cannabis use on adult psychosis by a functional polymorphism in the catechol-O-methyltransferase gene: longitudinal evidence of a gene X environment interaction. Biol Psychiatry. (2005) 57:1117–27. doi: 10.1016/j.biopsych.2005.01.026, PMID: 15866551

[57] Degenhardt L Tennant C Gilmour S. The temporal dynamics of relationships between cannabis, psychosis and depression among young adults with psychotic disorders: findings from a 10-month prospective study. Psychol Med. (2007) 37:927–34. doi: 10.1017/S0033291707009956, PMID: 17288638

[58] Hides L Dawe S Kavanagh DJ Young RM. Psychotic symptom and cannabis relapse in recent-onset psychosis. Prospective study. Br J Psychiatry. (2006) 189:137–43. doi: 10.1192/bjp.bp.105.014308, PMID: 16880483

[59] Stokes PR Egerton A Watson B Reid A Lappin J Howes OD. History of cannabis use is not associated with alterations in striatal dopamine D2/D3 receptor availability. J Psychopharmacol. (2012) 26:144–9. doi: 10.1177/0269881111414090 21890594

[60] Hirvonen J Goodwin RS Li CT Terry GE Zoghbi SS Morse C. Reversible and regionally selective downregulation of brain cannabinoid CB1 receptors in chronic daily cannabis smokers. Mol Psychiatry. (2012) 17:642–9. doi: 10.1038/mp.2011.82, PMID: 21747398 PMC3223558

[61] Bloomfield MA Morgan CJ Egerton A Kapur S Curran HV Howes OD. Dopaminergic function in cannabis users and its relationship to cannabis-induced psychotic symptoms. Biol Psychiatry. (2014) 75:470–8. doi: 10.1016/j.biopsych.2013.05.027, PMID: 23820822

[62] van de Giessen E Weinstein JJ Cassidy CM Haney M Dong Z Ghazzaoui R. Deficits in striatal dopamine release in cannabis dependence. Mol Psychiatry. (2017) 22:68–75. doi: 10.1038/mp.2016.21, PMID: 27001613 PMC5033654

[63] Volkow ND Wang GJ Telang F Fowler JS Alexoff D Logan J. Decreased dopamine brain reactivity in marijuana abusers is associated with negative emotionality and addiction severity. Proc Natl Acad Sci U S A. (2014) 111:E3149–56. doi: 10.1073/pnas.1411228111, PMID: 25024177 PMC4121778

[64] Leroy C Karila L Martinot JL Lukasiewicz M Duchesnay E Comtat C. Striatal and extrastriatal dopamine transporter in cannabis and tobacco addiction: a high-resolution PET study. Addict Biol. (2012) 17:981–90. doi: 10.1111/j.1369-1600.2011.00356.x, PMID: 21812871

[65] Albrecht DS Skosnik PD Vollmer JM Brumbaugh MS Perry KM Mock BH. Striatal D(2)/D(3) receptor availability is inversely correlated with cannabis consumption in chronic marijuana users. Drug and Alc Dep. (2013) 128:52–7. doi: 10.1016/j.drugalcdep.2012.07.016, PMID: 22909787 PMC3532956

[66] Farde L Wiesel FA Halldin C Sedvall G. Central D2 dopamine receptor occupancy in schizophrenic patients treated with antipsychotic drugs. Arc Gen Psychiatry. (1988) 45:71–6. doi: 10.1001/archpsyc.1988.01800250087012, PMID: 2892477

[67] Liechti ME Saur MR Gamma A Hell D Vollenweider FX. Psychological and physiological effects of MDMA ("ecstasy") after pretreatment with the 5-HT(2) antagonist ketanserin in healthy humans. Neuropsychopharmacology. (2000) 23:396–404. doi: 10.1016/S0893-133X(00)00126-3, PMID: 10989266

[68] Martinotti G Miuli A Stigliano G Stigliano G Pettorruso M di Giannantonio M. Transcranial magnetic stimulation to treat substance use disorders and behavioral addictions: the state of the art. Evid Based Psychiatric Care. (2021) 7:40–6. doi: 10.36180/2421-4469-2021-7

[69] Ricci V Ceci F Di Carlo F Lalli A Ciavoni L Mosca A. Cannabis use disorder and dissociation: a report from a prospective first-episode psychosis study. Drug Alcohol Depend. (2021) 229:109118. doi: 10.1016/j.drugalcdep.2021.109118, PMID: 34688166

[70] Ricci V Ceci F Di Carlo F Di Muzio I Ciavoni L Santangelo M. First episode psychosis with and without the use of cannabis and synthetic cannabinoids: psychopathology, global functioning and suicidal ideation and antipsychotic effectiveness. Psychiatry Res. (2023) 320:115053. doi: 10.1016/j.psychres.2023.115053, PMID: 36682093

[71] Martinotti G De Risio L Vannini C Schifano F Pettorruso M Di Giannantonio M. Substance-related exogenous psychosis: a postmodern syndrome. CNS Spectr. (2021) 26:84–91. doi: 10.1017/S1092852920001479, PMID: 32580808

